# Molecular Surveillance of Multidrug-Resistant Bacteria among Refugees from Afghanistan in 2 US Military Hospitals during Operation Allies Refuge, 2021

**DOI:** 10.3201/eid3014.240152

**Published:** 2024-11

**Authors:** Cole Anderson, Francois Lebreton, Emma Mills, Brendan Jones, Melissa Martin, Hunter Smith, Roseanne Ressner, Sara Robinson, Wesley Campbell, Jason Smedberg, Michael Backlund, Diane Homeyer, Joshua Hawley-Molloy, Natalie Khan, Henry Dao, Patrick McGann, Jason Bennett

**Affiliations:** Landstuhl Regional Medical Center Landstuhl, Germany (C. Anderson, J. Smedberg, J. Hawley-Molloy, N. Khan, Henry Dao); Walter Reed Army Institute of Research, Silver Spring, Maryland, USA (F. Lebreton, E. Mills, B. Jones, M. Martin, P. McGann, J. Bennett); Global Emerging Infections Surveillance Branch, Armed Forces Health Surveillance Division, Silver Spring (H. Smith); Walter Reed National Military Medical Center, Bethesda, Maryland, USA (R. Ressner, S. Robinson, W. Campbell, M. Backlund); Brooke Army Medical Center, San Antonio, Texas, USA (D. Homeyer)

**Keywords:** antimicrobial resistance, bacteria, infection control, military medicine, global health, refugees, Afghanistan

## Abstract

In 2021, two US military hospitals, Landstuhl Regional Medical Center in Landstuhl, Germany, and Walter Reed National Military Medical Center (WRNMMC) in Bethesda, Maryland, USA, observed a high prevalence of multidrug-resistant bacteria among refugees evacuated from Afghanistan during Operation Allies Refuge. Multidrug-resistant isolates collected from 80 patients carried an array of antimicrobial resistance genes, including carbapenemases (*bla*_NDM-1_, *bla*_NDM-5_, *and bla*_OXA-23_) and 16S methyltransferases (*rmtC* and *rmtF*). Considering the rising transmission of antimicrobial resistance and unprecedented population displacement globally, these data are a reminder of the need for robust infection control measures and surveillance.

Antimicrobials are among the most widely used drugs worldwide and are essential for treating infections. However, antimicrobial drug effectiveness depends on the susceptibility of the targeted pathogens. The emergence of widespread antimicrobial resistance (AMR) among pathogens is limiting the use of these essential drugs and is a major threat to global health. An estimated 5 million deaths are associated with drug-resistant infections annually; without intervention, that number could increase to 10 million by 2050 (*1*).

The wars in Iraq and Afghanistan have highlighted the prevalence of AMR across the Middle East and South Asia. Isolation of multidrug-resistant (MDR) bacteria was common in combat-related infections, such as wound infections. Many of those infections were caused by ESKAPE pathogens (*Enterococcus faecium*, *Staphylococcus aureus*, *Klebsiella pneumoniae*, *Acinetobacter baumannii*, *Pseudomonas aeruginosa*, and *Enterobacter* spp.) and had varying levels of resistance and potential for nosocomial spread (*2*–*6*). 

In 2009, the Multidrug-Resistant Organism Repository and Surveillance Network (MRSN) at the Walter Reed Army Institute of Research (Bethesda, Maryland, USA) was established to combat AMR emergence within the US military healthcare system. The MRSN serves as the primary surveillance organization for MDR bacteria across the Department of Defense (DoD). Since 2009, the MRSN has collected >120,000 bacterial isolates from patients treated at military hospitals across the world. Together, the MRSN and the DoD Global Emerging Infections Surveillance Branch coordinate a worldwide network of AMR surveillance across both military treatment facilities and partner nation settings.

In May 2021, the Taliban in Afghanistan began a military offensive that led to the fall of the Islamic Republic of Afghanistan 3 months later. More than 500,000 citizens of Afghanistan were displaced in 2021 and joined >5 million other refugees who have been displaced throughout the world during the past 2 decades. In July 2021, the US State Department established Operation Allies Refuge (OAR) to support a special immigrant visa program for eligible Afghanistan nationals who assisted the US government during the war, along with their families. On August 26, 2021, a suicide bombing at Hamid Karzai International Airport in Kabul, Afghanistan, prompted emergent medical evacuation of injured Afghanistan civilians to Landstuhl Regional Medical Center (LRMC; Landstuhl, Germany) and Walter Reed National Military Medical Center (WRNMMC; Bethesda, Maryland, USA). MRSN performed genomic evaluation on MDR bacteria isolated from evacuated patients at LRMC and WRNMMC. We assessed genomic relatedness from AMR isolates among OAR patients and historical strains.

## Methods

According to institutional infection control policy, perirectal and nasal surveillance swab samples were collected on all patients from Afghanistan at admission to LRMC and WRNMMC. Swab samples were used to screen for methicillin-resistant *Staphylococcus aureus* (MRSA), extended spectrum β-lactamase (ESBL)–producing bacteria, carbapenemase-producing *Enterobacterales* (CPE), and vancomycin-resistant *Enterococcus* (VRE). We defined an MDR organism (MDRO) as MRSA, VRE, CPE, or isolates recovered from clinical or surveillance cultures that were resistant to >1 agent in 3 different antimicrobial classes. We considered recovered isolates that met those criteria surveillance culture to be positive. Patients with negative surveillance cultures were immediately released from isolation; patients with positive surveillance or clinical cultures remained in isolation and on contact precautions to prevent nosocomial transmission. Isolates from all MDROs were sent to the MRSN for whole-genome sequencing. During September–October 2021, WRNMMC and LRMC submitted 171 MDR bacterial isolates from 80 inpatients and outpatients from Afghanistan. We used Illumina Miseq and MiSeq Reagent Kit version 3 (Illumina, https://www.illumina.com) for sequencing (600 cycles, 2 × 300 bp).

## Results

**Figure 1:**
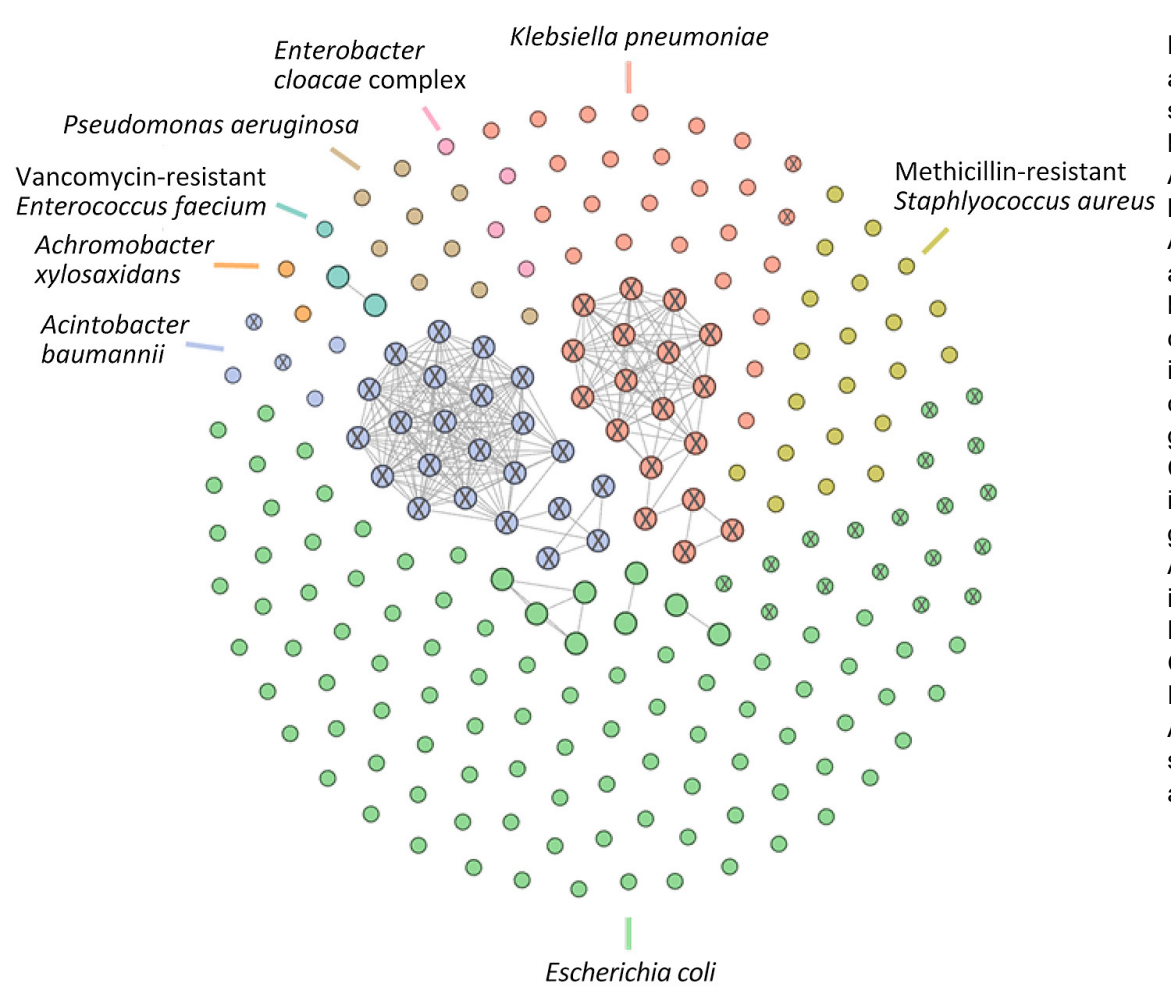
Cluster network of all isolates from molecular surveillance of multidrug-resistant bacteria among refugees from Afghanistan in 2 US military hospitals during Operation Allies Refuge, 2021. Isolates are grouped and colored by bacterial species. X indicates carbapenemase-producing isolates. Circles without connecting lines represent genetically unrelated isolates. Connected circles represent isolates that are <10 core alleles genetically distinct from Operation Allies Refuge isolates or historical isolates from Landstuhl Regional Medical Center, Landstuhl, Germany and Bagram Air Force Base, Parwan Province, Afghanistan. Clusters grouping serial isolates from single patients are not shown.

Among 80 patients with MDR isolates, 42 were male and 38 were female, and their median age was 26 years (range 1 week to 83 years). The top 5 clinical diagnoses were blast trauma (28%; n = 22), gunshot wound (15%; n = 12), urinary tract infection (11%; n = 9), abscess (10%; n = 8), and pregnancy (6%; n = 5). Among the isolates analyzed, *E. coli* was the most prevalent (58%; n = 99) bacterial species, followed by *K. pneumoniae* (13%; n = 22), *S. aureus* (12%; n = 20), and *A. baumannii* complex (5%; n = 9) (Figure 1).

After removal of serial isolates from the same patient (same sequence-type determined by in silico multilocus sequence typing), we used the deduplicated dataset of 132 isolates from 80 patients to investigate the prevalence of species and antimicrobial resistance genes. Of the 132 deduplicated isolates, *E. coli* was the most prevalent (64%; n = 85), followed by *K. pneumoniae* (12%; n = 16), *S. aureus* (11%; n = 14), and *A. baumannii* (5%; n = 6) (Table 1). Of the 80 distinct patients, 59 were identified with solitary MDRO-positive surveillance cultures of *E. coli* (76%; n = 45), *Klebsiella* (7%; n = 4), and *Enterobacter* spp. (2%; n = 1). Nine patients were colonized with multiple species in addition to >1 strain of *E. coli*: 5 (9%) with *E. coli* and *K. pneumoniae*, 2 (3%) with *E. coli* and VRE, and 2 (3%) with *E. coli* and 3 additional species. Overall, we found 92% (n = 54) of colonized patients carried an ESBL-positive *E. coli*. At LRMC, 85% (n = 30; data not shown) of patients who received surveillance cultures were colonized with >1 MDRO.

**Table 1:** Sources of 132 deduplicated isolates collected during molecular surveillance of multidrug-resistant bacteria among refugees from Afghanistan in 2 US military hospitals during Operation Allies Refuge, 2021

Species	No. by patient source					Total
	Respiratory	Urine	Wound	Blood	Rectal swab	
*Achromobacter xylosoxidans*	0	0	1	0	0	1
*Acinetobacter baumannii*	2	0	4	0	0	6
*Enterobacter cloacae* complex	0	0	1	0	2	3
*Enterococcus faecium*	0	0	0	0	3	3
*Escherichia coli*	0	10	3	1	71	85
*Klebsiella pneumoniae*	0	3	3	0	10	16
*Pseudomonas aeruginosa*	0	1	1	0	2	4
*Staphylococcus aureus*	3	0	11	0	0	14
Total	5	14	24	1	88	132

Among deduplicated *E. coli* isolates, 84% (n = 71) were ESBL-positive, mostly due to *bla*_CTX-M-15_; 16% (n = 14) were carbapenemase-positive, including 9 isolates carrying *bla*_NDM-5_ metallo-β-lactamase. We noted high diversity among the 85 *E. coli* isolates and identified 45 distinct sequence types (STs). Lineages ST10 (8 patients) and ST69 (6 patients) were most common. We identified only 2 patients with extraintestinal pathogenic *E. coli* lineage ST131. A total of 15 patients carried 2 (n = 11) or 3 (n = 4) distinct *E. coli* lineages. A single isolate of ST38 was shown to coproduce New Delhi metallo-β-lactamase (NDM) 5 and oxacillinase (OXA) 181 carbapenemases (Table 2). 

**Table 2:** Distribution of high priority antimicrobial resistance genes among isolates collected for molecular surveillance of multidrug-resistant bacteria among refugees from Afghanistan in 2 US military hospitals during Operation Allies Refuge, 2021

Species	*bla*_NDM-1_	*bla*_NDM-5_	*bla*_OXA-23_	*bla*_OXA-943_	*bla*_OXA-181_	*bla*_OXA-232_	*vanA*	*mecA*	*rmtC*	*rmtF2*	*rmtF1*	*rmtB1*
*Escherichia coli*	0	10	0	0	8	0	0	0	0	0	0	2
*Acinetobacter baumannii*	0	0	6	2	0	0	0	0	0	0	0	0
*Klebsiella pneumoniae*	1	0	0	0	1	3	0	0	1	1	2	0
*Enterococcus faecium*	0	0	0	0	0	0	3	0	0	0	0	0
*Staphylococcus aureus*	0	0	0	0	0	0	0	14	0	0	0	0
Total	1	10	6	2	9	3	3	14	1	1	1	2

We identified 16 distinct lineages among the 15 patients carrying *K. pneumoniae*. Overall, 81% (n = 13) of *K. pneumoniae* isolates carried *bla*_CTX-M-15_, and 19% (n = 3) were carbapenemase-positive due to *bla*_NDM-1_, *bla*_OXA-181_, and *bla*_OXA-232_. 

Among other identified MDRO isolates, we identified various antimicrobial resistance genes. Of the 6 patients with *A. baumannii*–positive cultures, 4 carried the epidemic clone ST2 that had *bla*_OXA-23_ carbapenemase. Two of the *E. cloacae* isolates carried *bla*_CTX-M-15_. All 14 *S. aureus* isolates carried the *mecA* gene, and all 3 *E. faecium* isolates carried the *vanA* gene. None of the 4 *P. aeruginosa* isolates carried high-risk AMR genes.

**Figure 2:**
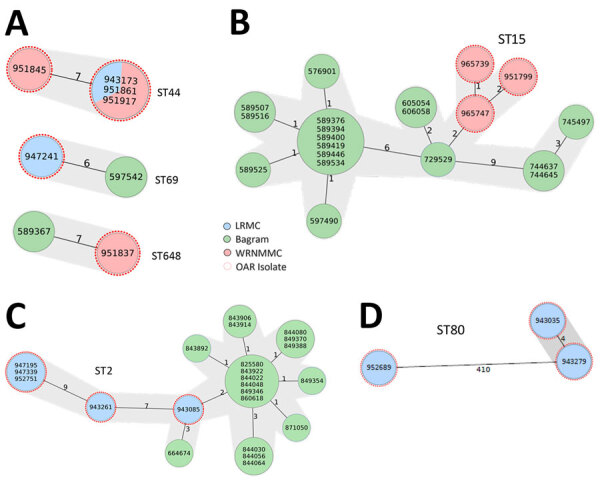
Phylogenetic analysis of highly related isolates from molecular surveillance of multidrug-resistant bacteria among refugees from Afghanistan in 2 US military hospitals during OAR, 2021. A) *Escherichia coli* ST44, ST69, and ST648 isolates. B) *Klebsiella pneumoniae* ST15 isolates; OAR isolates were closely related to isolates collected in 2018. C) *Acinetobacter baumannii* ST2 isolates; OAR isolates were closely related to isolates collected during 2019–2020. D) *Enterococcus faecium* ST80 isolates. Numbers inside circles indicate isolate identification numbers. Numbers along lines connecting circles indicate the number of allelic differences between isolates. Gray shading indicates clustered isolates. Bagram, Bagram Air Force Base, Bagram, Afghanistan; LRMC, Landstuhl Regional Medical Center, Landstuhl, Germany; OAR, Operation Allies Refuge; ST, sequence type; WRNMMC, Walter Reed National Military Medical Center, Bethesda, Maryland, USA.

To identify clusters of high genetic relatedness, we compared genomes of all isolates from this study to historical isolates unrelated to OAR that were collected from LRMC and Bagram Air Force Base (AFB). Bagram AFB was located in the northeastern province of Parwan in Afghanistan, 40 km north of Kabul, and was evacuated by US military personnel in July 2021. Bagram AFB housed the Craig Joint Theater Hospital that provided healthcare to US and coalition forces. We identified a total of 6 genetic clusters: 3 *E. coli*, and 1 cluster each of *K. pneumoniae*, *A. baumannii*, and *E. faecium* (Figure 2). We detected 3 clusters of epidemic *E. coli*, ST44, ST69, and ST648 (Figure 2, panel A). The ST44 cluster contained isolates from 2 OAR patients whose admissions overlapped at WRNMMC; those isolates differed by 14­–15 single-nucleotide polymorphisms (SNPs). The ST69 and ST648 clusters comprised 1 OAR patient from 2021 and 1 Bagram AFB patient whose isolates were collected in 2018; isolates in those clusters differed by 30 SNPs. 

In the *K. pneumoniae* cluster, 3 serial ST15 isolates that carried *bla*_OXA-232_, *bla*_CTX-M-15_, and *rmtF* (16S rRNA methyltransferase) were isolated from a single OAR patient at WRNMMC. Those isolates were highly related (17–45 SNPs difference) to isolates previously collected from 5 patients at Bagram AFB in 2018 (Figure 2, panel B). That OAR patient was also colonized with VRE and *E. coli* ST38 and ST648. In the *A. baumannii* cluster, 3 OAR patients from LRMC carried ST2 isolates that were highly related to a protracted nosocomial outbreak at Bagram AFB during 2019–2020 (Figure 2, panel C), and differed only by 11–38 SNPs. Last, 3 patients in the *E. faecium* cluster each had an isolate belonging to the ST80 epidemic clone (Figure 2, panel D). Two of those *E. faecium* isolates only differed by 7 SNPs from 2 patients in isolation at the same time at LRMC. Those patients were wounded in Afghanistan 2–3 weeks before evacuation to LRMC and were admitted 1 day apart from each other. 

Among the *S. aureus* isolates, we identified ST1482 in 43% (n = 6) of patients; the other patients carried clones ST30 (n = 4), ST22 (n = 3), and ST772 (n = 1). Although many patients shared the same ST, we did not identify any clusters of isolates sharing high levels of genetic relatedness.

## Discussion

In this study, MDRO surveillance was conducted at 2 US military hospitals that provided medical care to refugees from Afghanistan during OAR. We observed high rates of MDRO colonization with *E. coli* and found that many isolates carried ESBL, carbapenemase, and 16S methyltransferase genes. Furthermore, we observed many AMR *K. pneumoniae*, *A. baumannii*, *E. faecium*, and *S. aureus* clinical isolates. We found that >75% of ESBL-producing *E. coli* isolates produced CTX-M-15. Among the 14 carbapenemase-producing *E. coli* isolates, we isolated *E. coli* ST648 coproducing NDM-5 and RmtB in samples from 5 patients. ST648 has been characterized by a combination of multidrug resistance, high-level virulence, and biofilm formation, similar to the global high-risk ST131 clonal lineage (*7*). One patient was colonized with *K. pneumoniae* ST11 that coproduced NDM-1 and RmtC, a lineage that is commonly known for high virulence and resistance to all β-lactams, including carbapenems, and resistance to aminoglycosides (*8*).

One patient from WRNMMC had 3 serial *K. pneumoniae* ST15 isolates from an abdominal wound and surveillance cultures. Surveillance cultures from that patient were also positive for VRE, *P. aeruginosa*, and 2 ESBL-producing strains of *E. coli*, ST38 and ST648. In addition to *bla*_OXA-232_, *bla*_CTX-M-15_, and *rmtF*, the *K. pneumoniae* ST15 isolates carried markers of hypervirulence and hypermucoviscosity (data not shown). The siderophores, yersiniabactin and aerobactin, *rmpA2* (regulator of mucoid phenotype), and *peg344* (a drug and metabolite transporter) were detected in those isolates, and all of those have been associated with invasive disease in immunocompetent patients (*9*). OXA-232–producing *K. pneumoniae* ST15 predominantly is found in China, where multiple nosocomial outbreaks have been reported, but the lineage also has been described globally (*10*).

Nosocomial outbreaks causing severe illness and death have been reported in medical treatment facilities in Iraq and Afghanistan and are a great concern for the US military healthcare system (*11*). Although phylogenetic analysis in this study revealed multiple clusters of highly related isolates, patients in those clusters were likely colonized or infected before entering US military hospitals. Prevalence of high-risk AMR genes is high in Afghanistan, and patients could have acquired environmental MDRO; however, those possibilities would be difficult to determine because microbiologic sampling was conducted after evacuation to LRMC. Alternatively, patients could have acquired MDR bacteria during hospitalization in Afghanistan before evacuation. However, we do not know the extent of medical treatment patients received in Kabul. 

The *A. baumannii* ST2 isolates collected during this study were genetically related (11–38 SNPs) to nosocomial ventilator-associated pneumonia and bloodstream infections collected at Bagram AFB during 2019–2020 (C. Anderson et al., unpub. data). Two clusters were identified in that outbreak, comprising 10 patients from Afghanistan with *A. baumannii* ST2. The 2 clusters containing ST2 were separated by 80 allelic differences, which suggests 2 distinct strains of ST2 were involved in nosocomial transmission. Isolates within those clusters were found to differ by only 3–13 SNPs in 1 cluster and 1–4 SNPs in the other. Patients in that outbreak were transferred to Bagram AFB after initial treatment in hospitals in Afghanistan, but an outbreak investigation at Bagram AFB did identify lapses in infection control protocols that could have led to nosocomial transmission within those clusters after independent introductions of the strains from hospitals in Afghanistan. Despite the high prevalence of MDROs observed during OAR and the genetic similarities between some of the isolates, we found no evidence to suggest nosocomial spread occurred at LRMC or WRNMMC. That point highlights how crucial rapid screening, patient cohorting, and preemptive isolation upon hospital admission are to preventing nosocomial outbreaks within the military healthcare system.

We observed high rates of MDRO colonization in this study. Risk factors for MDRO acquisition include international travel, travel-related diarrhea, antibiotic use, and prolonged hospitalization (*12*). Among military personnel, Afghanistan-based personnel have been found to have a 5.5-fold higher prevalence of MDR *E. coli* colonization than US-based personnel (*13*). At LRMC, >80% of patients from Afghanistan had a positive MDRO surveillance culture, much higher than the 30% rate among US patients during the same timeframe (data not shown). Those rates are consistent with studies that describe high MDRO colonization rates or MDRO outbreaks among refugees from regions with high AMR prevalence. A 2016 study in Germany observed an MDRO prevalence in hospital-admitted refugees of up to 60.8%, and a preponderance of ESBL-producing *Enterobacterales* (*14*). Of note, >50% of the refugees in this study were from Syria or Afghanistan, and only 16.7% of nonrefugee patients were found to be positive for MRDOs. Similar observations in Germany, such as increased reports of NDM-1– producing *K. pneumoniae* in refugees and injured soldiers evacuated from Ukraine, have also been reported (*15*). Of note, prior hospitalization in Ukraine is now considered a major risk factor for MDRO colonization, because infection control measures have become inadequate due to limited resources and personnel since the 2022 invasion by Russia.

Although this study provides a unique snapshot of the AMR burden in Afghanistan, our results are limited by the small sample size and sampling bias because data were only available for refugees who were evacuated from Kabul and received care at LRMC or WRNMMC. This study also lacked detailed medical history before medical evacuation, such as the extent of hospitalization in Kabul. Further, microbiologic sampling was conducted after patients were evacuated from Afghanistan. Therefore, whether MDRO acquisition occurred during hospitalization or within the community environment because of high AMR prevalence in this region cannot be determined.

In summary, this study describes a high prevalence of MDRO among refugees from Afghanistan evacuated to US military hospitals during OAR. Molecular surveillance identified multiple high-risk clonal lineages that were characterized by extensive AMR and hypervirulence profiles and were genetically related to historical isolates collected during the war in Afghanistan. Unlike other reports that focused primarily on war wounds among military personnel injured in Afghanistan, this study uniquely provides detailed genomic AMR data, from both clinical and surveillance isolates, on refugees from Afghanistan who received care within the US military healthcare system. When viewed in the context of rising global AMR transmission and unprecedented population displacement, these data are a reminder of how crucial robust infection control measures and surveillance are to protecting public health.
